# Preferences of patients and surgeons regarding counselling before pancreatectomy: 4PC trial

**DOI:** 10.1093/bjsopen/zrae128

**Published:** 2024-10-22

**Authors:** Antonie Willner, Olga Radulova-Mauersberger, Anuschka Barenbrock, Marius Distler, Sandra Korn, Rolidy Jimenez, Mara R Goetz, F Guentac Uzunoglu, Tina Groß, Benjamin Muessle, Thilo Hackert, Juergen Weitz, Thilo Welsch

**Affiliations:** Klinik für Allgemein-, Viszeral- und Thoraxchirurgie, Universitätsklinikum Hamburg-Eppendorf, Hamburg, Germany; Klinik für Allgemein-, Viszeral- und Thoraxchirurgie, Oberschwabenklinik Ravensburg, Akademisches Lehrkrankenhaus der Universität Ulm, Ravensburg, Germany; Klinik für Viszeral-, Thorax- und Gefäßchirurgie, Universitätsklinikum Carl Gustav Carus Dresden, Dresden, Germany; Klinik für Viszeral-, Thorax- und Gefäßchirurgie, Universitätsklinikum Carl Gustav Carus Dresden, Dresden, Germany; Klinik für Viszeral-, Thorax- und Gefäßchirurgie, Universitätsklinikum Carl Gustav Carus Dresden, Dresden, Germany; Klinik für Viszeral-, Thorax- und Gefäßchirurgie, Universitätsklinikum Carl Gustav Carus Dresden, Dresden, Germany; Klinik für Viszeral-, Thorax- und Gefäßchirurgie, Universitätsklinikum Carl Gustav Carus Dresden, Dresden, Germany; Klinik für Allgemein-, Viszeral- und Thoraxchirurgie, Universitätsklinikum Hamburg-Eppendorf, Hamburg, Germany; Klinik für Allgemein-, Viszeral- und Thoraxchirurgie, Universitätsklinikum Hamburg-Eppendorf, Hamburg, Germany; Klinik für Allgemein-, Viszeral- und Thoraxchirurgie, Universitätsklinikum Hamburg-Eppendorf, Hamburg, Germany; Klinik für Allgemein-, Viszeral- und Thoraxchirurgie, Oberschwabenklinik Ravensburg, Akademisches Lehrkrankenhaus der Universität Ulm, Ravensburg, Germany; Klinik für Allgemein-, Viszeral- und Thoraxchirurgie, Oberschwabenklinik Ravensburg, Akademisches Lehrkrankenhaus der Universität Ulm, Ravensburg, Germany; Klinik für Allgemeine und Viszerale Chirurgie, Universitätsklinikum Ulm, Ulm, Germany; Klinik für Allgemein-, Viszeral- und Thoraxchirurgie, Universitätsklinikum Hamburg-Eppendorf, Hamburg, Germany; Klinik für Viszeral-, Thorax- und Gefäßchirurgie, Universitätsklinikum Carl Gustav Carus Dresden, Dresden, Germany; Klinik für Allgemein-, Viszeral- und Thoraxchirurgie, Universitätsklinikum Hamburg-Eppendorf, Hamburg, Germany; Klinik für Allgemein-, Viszeral- und Thoraxchirurgie, Oberschwabenklinik Ravensburg, Akademisches Lehrkrankenhaus der Universität Ulm, Ravensburg, Germany; Medizinische Fakultät, Technische Universität Dresden, Dresden, Germany

Postoperative morbidity after pancreatic resection may negatively impact patients’ quality of life and life expectancy^1,2^. This underscores the importance of the items that are mentioned when obtaining informed consent and during counselling. There is evidence that counselling may reduce fear and anxiety, as well as promote patient recovery^3^. The aim of this study was to compare patients’ preferences regarding detailed informed consent and counselling items before pancreatic resection with the respective surgeons’ preferences and with their postoperative preferences.

To this end, a prospective multicentre survey including 53 items was conducted at three German certified academic pancreatic centres. Patients who underwent elective pancreatic resection between August 2022 and August 2023 were surveyed twice (before and after surgery). Surgeons were surveyed once using the same questionnaire. Study participants were asked to rate each single item on a five-point Likert scale and to rank six subcategories of items. Additional information can be found in the *Supplementary Methods*.

A total of 83 patients (of 105 patients enrolled) and 45 surgeons were analysed (*Fig. S1*). The most common resections were pancreatoduodenectomy (45 patients; 54%) and distal pancreatectomy (18 patients; 21.7%). The characteristics of the patients and surgeons are provided in *Tables S2*, *S3*.

Among the six subcategories of all survey items, patients considered the quality criteria of the surgical centre, including hospital volume and surgeon expertise (mean(s.d.) 2.88(1.87)), to be most important in the preoperative setting, followed by the surgical technique (mean(s.d.) 2.90(1.73)) (*Fig. 1*). In contrast, the description of the operation was considered to be the most important subcategory by the surgeons (mean(s.d.) 1.53(1.18)), whereas the subcategory concerning the quality criteria of the surgical centre was considered to be the least important (mean(s.d.) 5.24(1.23)). Whereas the largest mean differences in rank order between patients and surgeons were found for the quality criteria (subcategory ‘volume’, *P* < 0.001) and the description of the operation (*P* < 0.001), the third largest difference was observed for the subcategory ‘quality of life’, which was more important to patients than surgeons (mean rank 3.09 *versus* 4.20 respectively, *P* < 0.001). There were no significant changes in patient preferences in the postoperative setting, apart from surgical technique (such as organ preservation and minimally invasive or open approach), which was considered less important (*P* = 0.024) (*Figs S2*, *S4*).

**Fig. 1:**
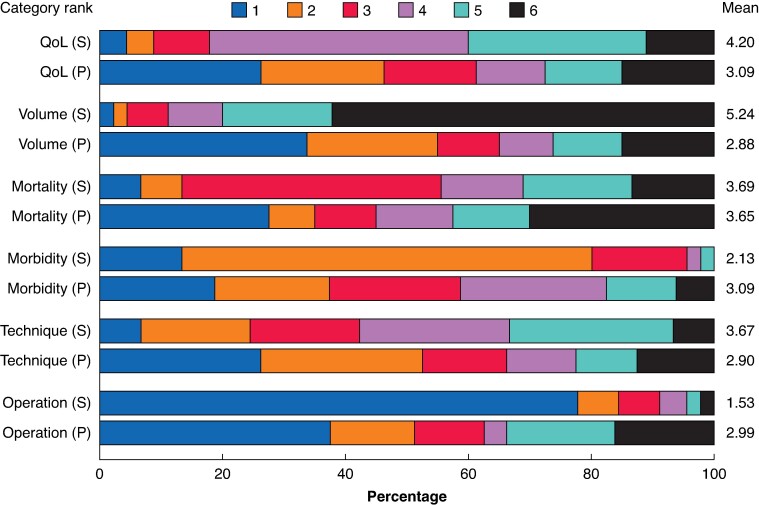
Survey results for patients (before surgery) and surgeons indicating the rank order of the six subcategories of items Patients and surgeons were independently asked to rank the following six subcategories from most important (1) to least important (6): ‘operation’, description of the operation, its course, and alternatives; ‘technique’, discussion of technical details, such as pylorus or spleen preservation, and laparoscopic, robotic, or open approach; ‘morbidity’, discussion of postoperative complications and potential reasons for an increased duration of hospital stay; ‘mortality’, discussion of the possibility of death during or after the operation; ‘volume’, discussion of quality criteria of the centre, including hospital volume and surgeon expertise; and ‘quality of life’, discussion of what to expect regarding quality of life after surgery, such as changes to diet, development of diabetes mellitus, and initiation of chemotherapy. The mean rank orders are indicated on the right-hand side. The mean rank of all subcategories, except ‘mortality’, differed significantly between patients and surgeons. S, ranking by 45 surgeons; P, preoperative ranking by 83 patients; QoL, quality of life.

The single items that were more essential for the patients (with a mean difference greater than or equal to 1.0 on the Likert scale) concerned surgeon volume, surgery being performed by a highly experienced surgeon, ward design (that is patients learning what the hospital ward and postoperative care unit/rooms look like), infections, and duration of chemotherapy (*Fig. S3*). A video of the surgical intervention was considered to be relevant, important, or extremely important by 34 (41%) of the patients, but only by 7 (16%) of the surgeons. A detailed analysis revealed further differences with regard to patient sex and other patient subgroups (*Supplementary Results*).

In the present trial, it is shown that patients highly appreciate preoperative information on the quality of the pancreatic centre (including hospital volume and surgeon expertise) and on quality-of-life aspects, rather than on the mortality rate. Different results have been reported after various gastrointestinal surgeries, with greater than 95% of patients indicating that the risk of death is extremely important to discuss^4^. A discrete-choice experiment revealed that discussion of quality of life is more important to patients during counselling before oesophagogastric surgery than hospital type and surgeon experience^5^. Compared with patients, surgeons place more emphasis on the indications for surgery, alternative approaches, and morbidity.

Surgeons may better integrate patients’ preferences and optimize preoperative counselling with information on hospital volume, surgeon expertise, and quality-of-life expectations. On the other hand, the study indicates that patients may underestimate the importance of the morbidity and mortality associated with pancreatic surgery.

In conclusion, the present study provides current patient preferences for preoperative counselling before pancreatic resection. Optimized and individualized counselling may improve preparedness and recovery.

## Supplementary Material

zrae128_Supplementary_Data

## Data Availability

The raw data supporting the findings are available from the corresponding author upon reasonable request.
