# Supplementary material for: Preferences of patients and surgeons regarding counselling before pancreatectomy: 4PC trial
Source: BJS Open. 2024 Oct 22;8(5):zrae128. doi: 10.1093/bjsopen/zrae128 (PMC11494370; doi:10.1093/bjsopen/zrae128)
Supplement: zrae128_Supplementary_Data [file zrae128_supplementary_data.docx]

**Preferences of patients and surgeons regarding preoperative counselling contents before pancreatectomy: 4PC trial**

Antonie Willner^1,3*^, Olga Radulova-Mauersberger^2*^, Anuschka Barenbrock^2^, Marius Distler^2^, Sandra Korn^2^, Mara Goetz^1^, F.G. Uzunoglu^1^, Tina Groß^1,3^, Benjamin Müssle^3,4^, Thilo Hackert^1^, Juergen Weitz^2^,Thilo Welsch^1,3,5^

^1^Klinik für Allgemein-, Viszeral- und Thoraxchirurgie, Universitätsklinikum Hamburg-Eppendorf, Hamburg

^2^Klinik für Viszeral-, Thorax- und Gefäßchirurgie, Universitätsklinikum Carl Gustav Carus Dresden, Dresden

^3^Klinik für Allgemein-, Viszeral- und Thoraxchirurgie, Oberschwabenklinik Ravensburg, Akademisches Lehrkrankenhaus der Universität Ulm

^4^Klinik für Allgemeine und Viszerale Chirurgie, Universitätsklinikum Ulm, Ulm

^5^Medizinische Fakultät, Technische Universität Dresden, Dresden

^*^contributed equally

**Corresponding author:**

Thilo Welsch, MD, FACS

Klinik für Allgemein-, Viszeral- und Thoraxchirurgie, Universitätsklinikum Hamburg-Eppendorf

Martinistr. 52, 20246 Hamburg, Germany

Mail: t.welsch@uke.de

**Supplementary Material - Index**

| **Supplementary Methods** | *pag. 3* |
| --- | --- |
| Study design, objects and ethical considerations | *pag. 3* |
| Patient and surgeon eligibility | *pag. 3* |
| Questionnaire | *pag. 4* |
| Statistical analysis and data presentation | *pag. 4* |
| **Supplementary Results** | *pag. 5* |
| Characterization of the study cohort, operations and morbidity | *pag. 5* |
| Detailed comparison of the patients’ pre- and postoperative survey and subgroup analysis | *pag. 5* |
| **Supplemental Appendixes** | *pag. 6* |
| Limitations of the study | *pag. 6* |
| **Supplemantary Figures and Tables** | *pag. 7* |
| Figure S1 | *pag. 7* |
| Figure S2 | *pag. 8* |
| Figure S3 | *pag. 9* |
| Figure S4 | *pag. 14* |
| Table S1 | *pag. 19* |
| Table S2 | *pag. 21* |
| Table S3 | *pag. 22* |
| **References** | *pag. 23* |

**Supplementary Methods**

Study design, objects and ethical considerations

The 4PC trial was a prospective observational multicentre survey study. The endpoints of the study were differences in patient- and surgeon-reported preferences and outcomes between combined and single counselling items. Furthermore, changes in postoperative preferences and the impact of patient and surgeon subgroups on preference and outcome ratings were investigated. The study was registered in advance (DRKS00030863). The study was approved by the respective ethics committees of all participating centres (Technische Universität Dresden/BO-EK-196042022, Landesärztekammer Baden-Württemberg/B-F-2022-059 and Landesärztekammer Hamburg/2023-200747-BO-bet).

Patient and surgeon eligibility

All patients were screened for eligibility considering the inclusion criteria in the outpatient department or on the day of admission. Patients who were scheduled for elective open or minimally invasive pancreatic resection for benign or malignant diseases of the pancreas, the distal bile duct or the duodenum were included. Further inclusion criteria were patients aged ≥18 years and an American Society of Anaesthesiology (ASA) score ≤III. After providing consent for the study, the patients were asked to complete the preoperative survey questionnaire before official counselling and the informed consent form to avoid systemic bias. The patients were asked again to complete identical surveys within three days of discharge from the hospital to evaluate changes during the postoperative course. Surgeons completed the same questionnaire once, irrespective of their position, surgical experience, or age.

Additionally, demographic data were collected and entered into a database created from medical records and surgeon office notes. Preoperative clinical characteristics, which could influence the outcome of surgical treatment, were recorded for each patient. These preoperative parameters included patient demographics, body mass index (BMI), diagnosis, previous pancreatic surgery and medical background knowledge. Surgical intervention and procedures as well as postoperative events, including morbidity (graded according to the Clavien‒Dindo classification of complications [CDC^1^] and length of hospital stay, were also considered. Morbidity was defined as all postoperative complications that occurred during the hospital stay until discharge. The trial included a total of four study visits extending from the outpatient consultation until the day of discharge from the hospital.

Patients who did not complete the pre- and postoperative questionnaire, who had a prolonged hospitalisation (>3 months prior to the index operation) or who died during this period were excluded from the final analysis. All surgeons at the three sites with previous experience in counselling or obtaining informed consent for pancreatic surgery were also invited to participate.

Questionnaire

The survey questionnaire was customised by the research team and evaluated as an anonymous pilot survey in ten patients who underwent abdominal surgery between January, 2022 and March, 2022 at one of the study centres. The intention of the pilot evaluation was to assess the feasibility and understandability of the different questions and items. The questionnaire was modified, and its final version was agreed upon.

The final survey questionnaire comprised seven pages and covered the following 6 subcategories with a total of 53 items (Table S1): (1) indication, timing and alternatives; (2) description of the operation technique, course and recovery; (3) postoperative morbidity and mortality; (4) quality and volume assessment of the centre and surgeons; (5) adjuvant treatment and rehabilitation options; and (6) postoperative limitations and quality of life (changes in quality of life, which are associated with dietary adjustments and the development of diabetes or the initiation of chemotherapy). Study participants were asked to rate each single item on a 5-point Likert scale (ranging from 1: "not relevant" to 5: "most important"). Additionally, participants were asked to rank the 6 subcategories in an order ranging from “1, most important” to “6, not relevant”.

Statistical analysis and data presentation

The survey was designed to include 80 patients and 40 surgeons. The survey should be representative of a population of 100 patients who were scheduled for an elective pancreas resection. To this end, the required sample size for completed survey questionnaires was 80, which was calculated using a confidence level of 95% and a margin of error of 5%. The sample size of the included surgeons was not calculated but was set to a minimum of 40. To compare the survey results, points based on the Likert scale for individual questions from all participants were added and tested as metric data using the Mann‒Whitney U test. Differences in patient survey outcomes pre- and postoperatively were analysed using the Wilcoxon test. A *P* value <0.05 was considered to indicate statistical significance. Population-based data are presented as median values and interquartile ranges (IQRs), and survey results are presented as the means ± standard deviations. Tests were performed, and graphs were generated using the R software package (version 3.1.3).

**Supplemental Results**

Characterisation of the study cohort, operations and morbidity

Of the 105 patients who were enrolled in the survey, 83 completed both surveys and were included in the final analysis (Figure S1). Regarding the survey among surgeons, the response rate was 41.7% (invited: n=108; analysed respondents: n=45). The median age of the patients was 66 years (IQR: 59–74 years) including 31 females (37.3%) and 52 males (62.7%) (Table S2). The median length of hospital stay was 16 days (IQR: 10–23), with morbidity (>3a) and mortality rates of 21.7% and 1.2% (one patient died within the hospital after the index operation, but was included because both, pre- and postoperative questionnaires had been completed before), respectively, among the analysed patients. The rate of clinically relevant POPF grade B or C was 15.7% (n=13), and PPH occurred in 7 patients (8.4%).

Twenty-seven percent of the surgeons were <35 years old. The percentage of consultant surgeons was 46.7% (n=21), whereas 22.2% held a chief position or were a programme director within the surgical department (n=10). One-third of the surgeons (n=15) had performed more than 20 pancreatic resections on their own (Table S3).

Detailed comparison of the patients’ pre- and postoperative survey and subgroup analyses

Among the 6 subcategories, changes in quality of life, which are associated with dietary adjustments and the development of diabetes or the initiation of chemotherapy, were most commonly (30.5%) placed on the top rank in the postoperative survey (Figures S2 and S3).

When pre- and postoperative preferences regarding all single survey items were compared, only 2 out of the 53 items were significantly different: alternatives to the surgical procedure were considered more important (P=0.027), and blood transfusion was less frequently preferred (P=0.001) postoperatively (Figure S4). A subgroup comparison of postoperative item rating between patients with and without major postoperative complications (CDC >3a) revealed no significant difference except for the item “ward design”. Patients with major complications placed more emphasis on the ward design compared with patients with no or minor complications (P=0.017).

There were some significant sex differences. In the preoperative setting, pain management seemed to be a more essential component for female patients (P=0.034), whereas males rated a second opinion (P=0.015), potential limitations in the ability to perform sports activities (P=0.027) and sexual function (P=0.002) as more important. Interestingly, further subgroup analysis did not reveal any significant differences in the surgeons’ ratings according to their surgical experience, position or age group.

**Supplementary Appendixes**

Limitations of the study

The study also has some limitations, including the fact that the survey may not be representative for all patients and surgeons because of the sample size or may be limited to the German population or medical system. Despite the fact that a pre-evaluation and modification of the questionnaire had been performed to improve the understandability before the start of the final survey, it cannot be excluded that individual patients may not had fully understood the nature of each survey item. The presented results may not characterise the subcohort of patients with an exceptionally prolonged duration of hospital stay (> 3 months) because of severe complications. This subgroup was excluded from the postoperative second survey and the study for ethical reasons. Some 23% of patients with severe complications (CDC grade >3a) were included in the final analysis, mitigating the argument that the study results are strongly biased by a highly selected subgroup with a non-complicated course.

**Supplementary Figures**

**Figure S1.** Study flow diagram

(A) Study flow diagram of patients included.

(B) Study flow diagram of surgeons included.

**Figure S2.** Results of the patients’ preoperative and postoperative survey indicating their rank order of the 6 subcategories of items.

Patients (n=83) were pre- and postoperatively asked to rank the following 6 subcategories from most important (1) to least important (6): “Operation”, description of the operation, its course and alternatives; “Technique”, technical details including pylorus or spleen preservation, laparoscopic, robotic or open approach; “Morbidity”, postoperative complications and consecutive prolongation of the hospital stay; “Mortality”, possibility to die from the operation; “Volume”, quality criteria of the centre and experience/volume of the centre and the surgeons with the operation; and “QoL”, postoperative expectations, e.g., dietary changes, diabetes mellitus, or chemotherapy. The mean rank order of the respective category and subcohort is indicated on the right of the plot.

(b) Ranking of patients before the operation; (a) Ranking of patients after the operation.

**
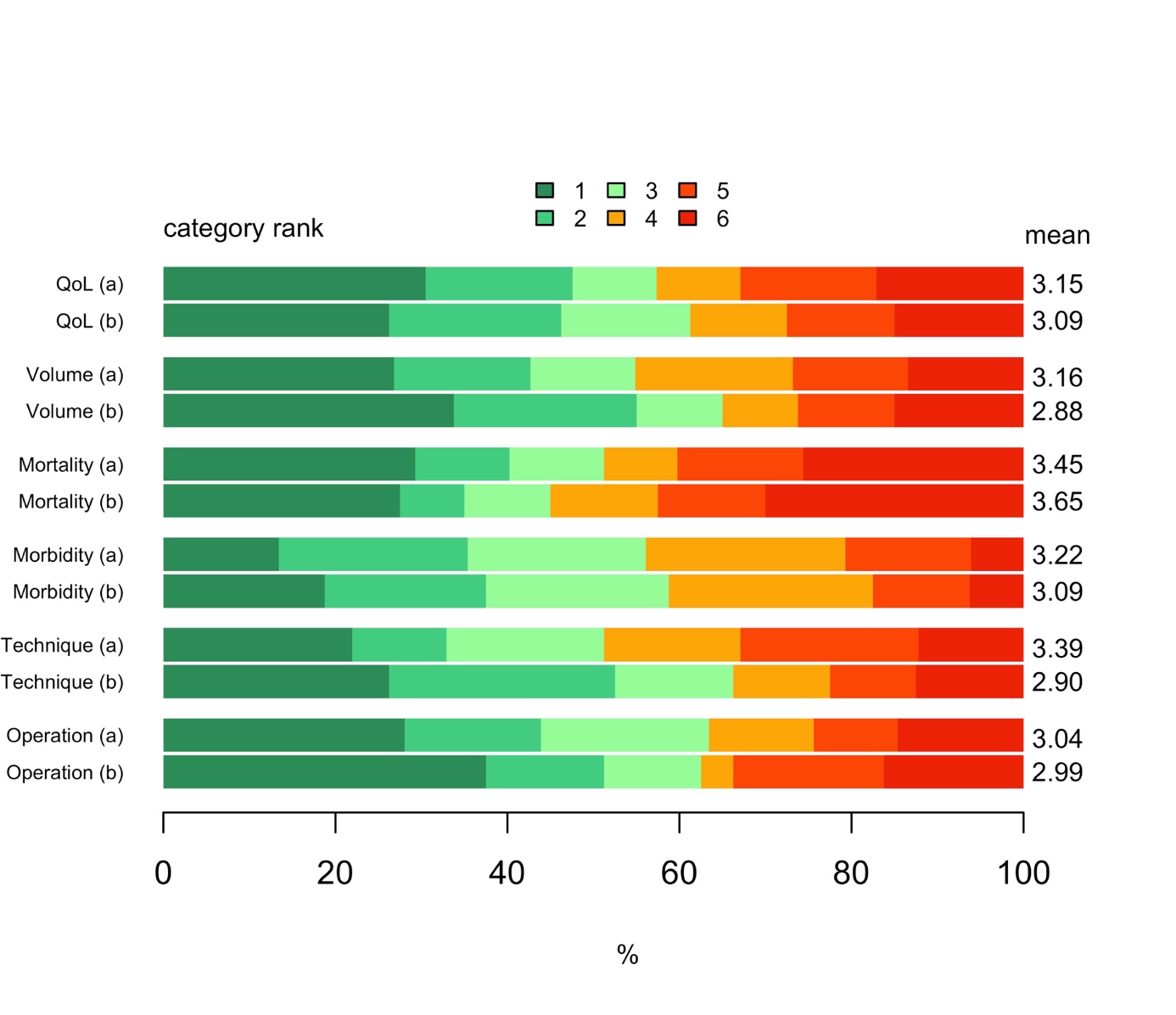
**

**Figure S3.** Survey results of single items compared between patients (preoperative) and surgeons

(A) Subcategory: Indication, timing and alternatives. *Abbreviations*: P, patients; S, surgeons; Indication, Timing, Alternatives, Prehab, Companion; refer to items nr. 1-5, Tab. S2.


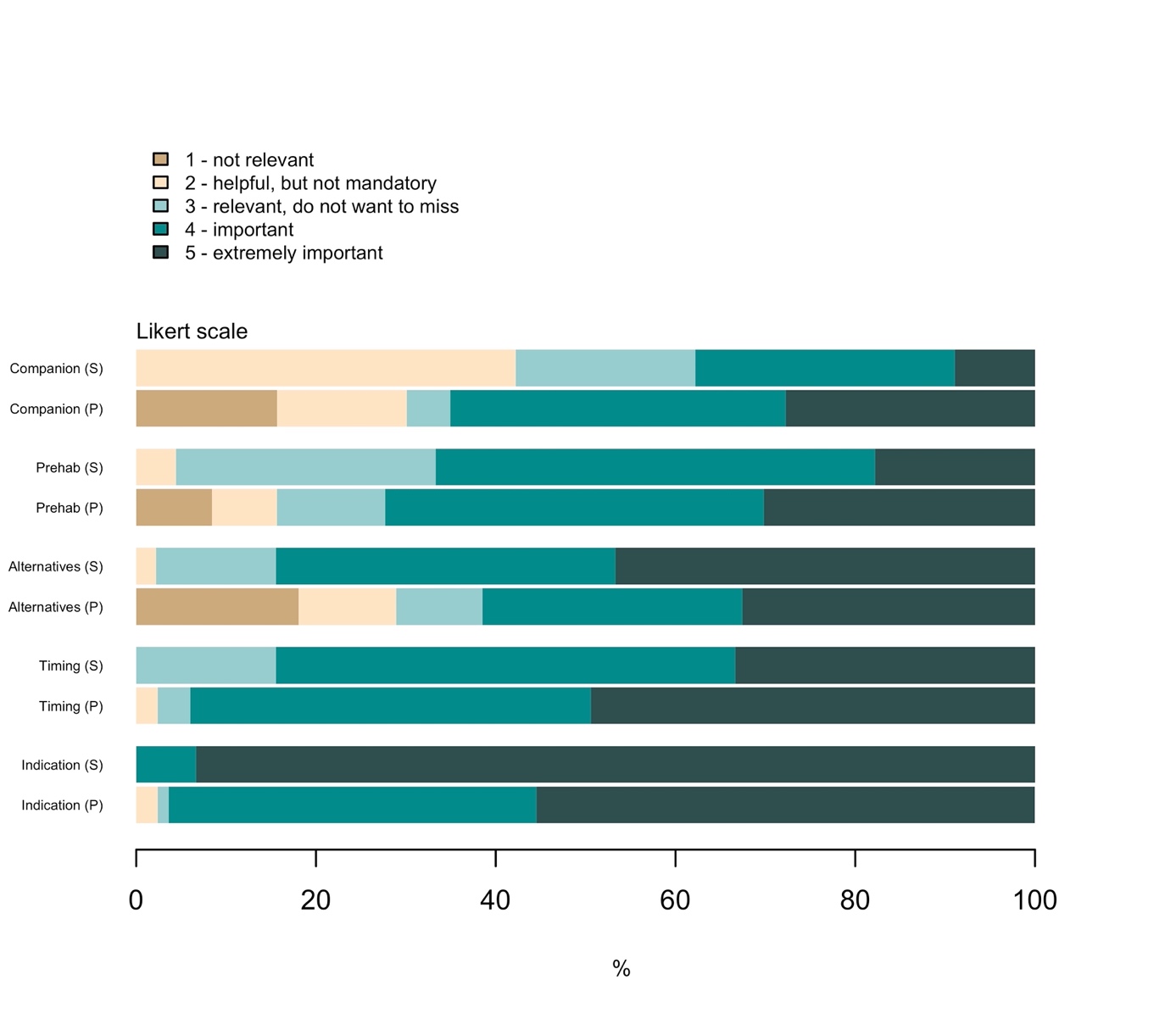


(B) Subcategory: Surgical intervention and procedure/ technique

*Abbreviations*: P, patients; S, surgeons; Anatomy, Drawing, Video, Approach, Preserving, Incision, Duration, Transfusion, Anaesthesia, Hospital stay, ICU stay, Nutrition, Mobilization, Ward design; refer to items nr. 6-19, Tab. S2.


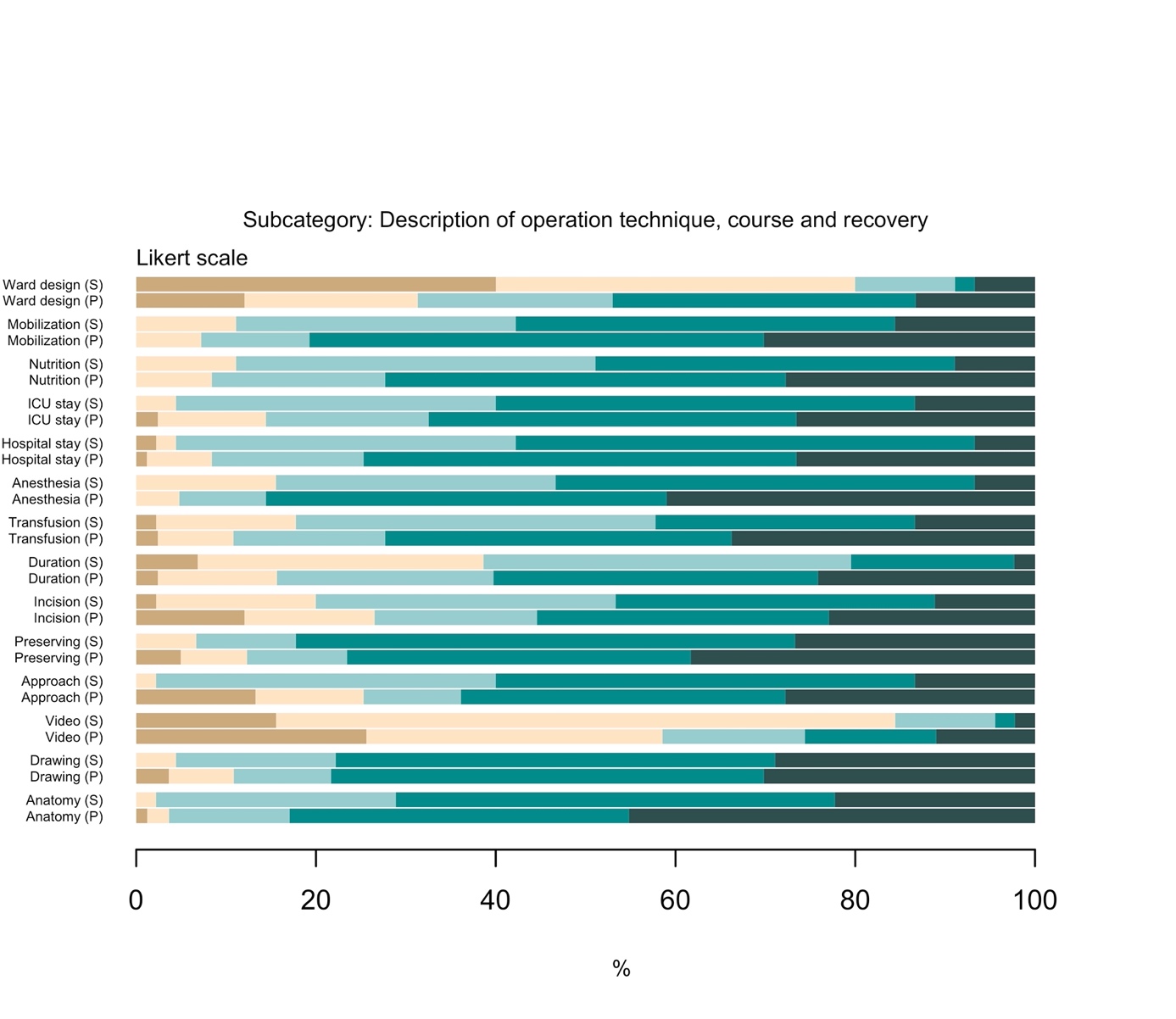


(C) Subcategory: Morbidity and mortality.

*Abbreviations*: P, patients; S, surgeons; Complications, Re-OP, Intervention, Infection, DVT/PE, NG tube, Pain, Intraop. problem, Stoma, MRGN/MRSA, Covid-19, Mortality; refer to items nr. 20-31, Tab. S2.


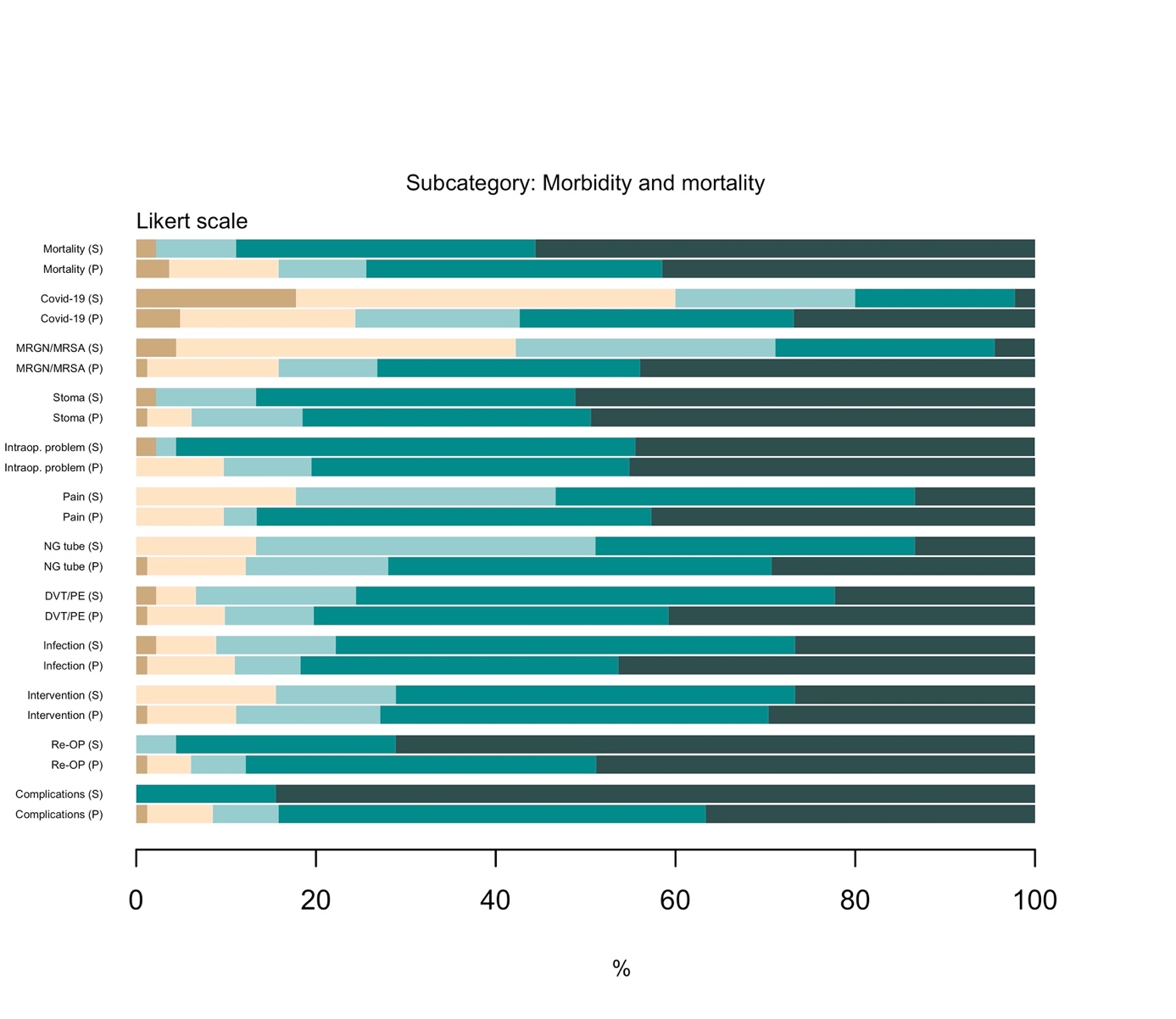


(D) Subcategory: Centre quality, volume and expertise.

*Abbreviations*: P, patients; S, surgeons; Centre expertise, Hospital volume, Surgeon volume, Certification, Senior surgeon, 2^nd^ opinion, Equipment; refer to items nr. 32-38, Tab. S2


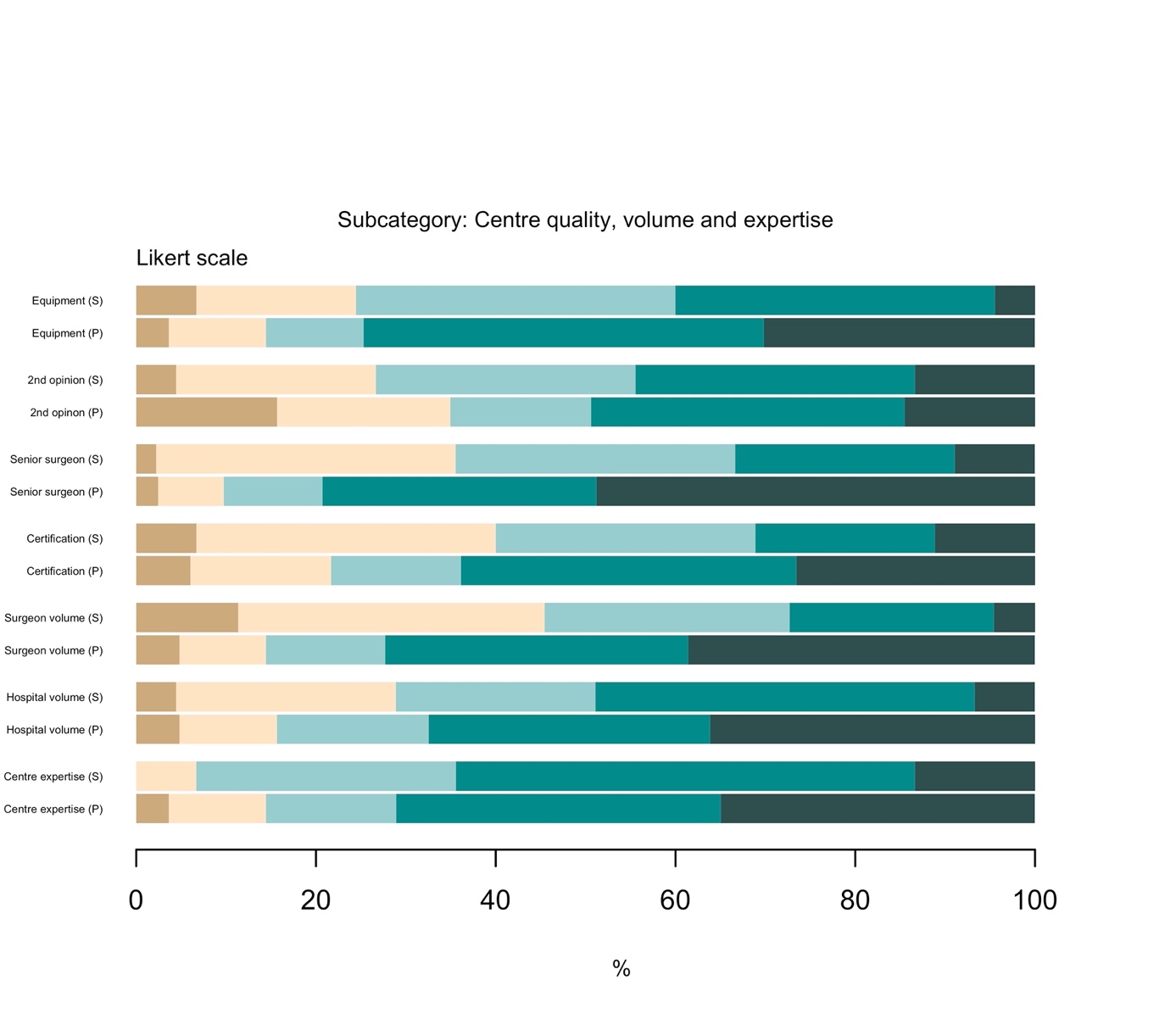


(E) Subcategory: Adjuvant treatment and rehabilitation.

*Abbreviations*: P, patients; S, surgeons; Possible chemo, Chemo effects, Chemo duration, Radiation, Rehabilitation; refer to items nr. 39-43, Tab. S2


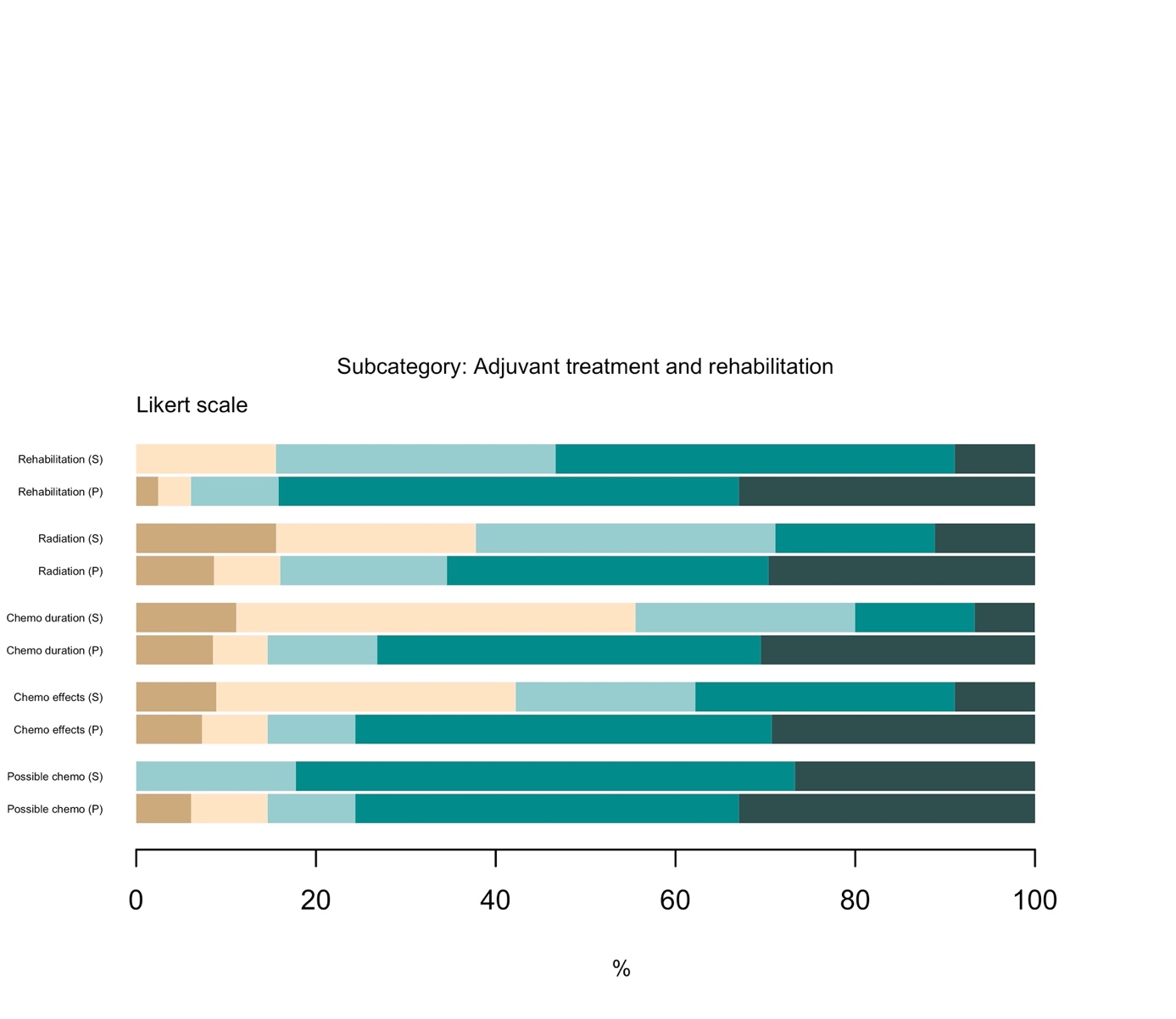


(F) Subcategory: Postoperative limitations and quality of life.

*Abbreviations*: P, patients; S, surgeons; Nutrition, Medication, Body weight, Diabetes, Back to work, Sports activity, Cosmetics; Sexual function; Daily care, Chances for cure; refer to items nr. 44-53, Tab. S2


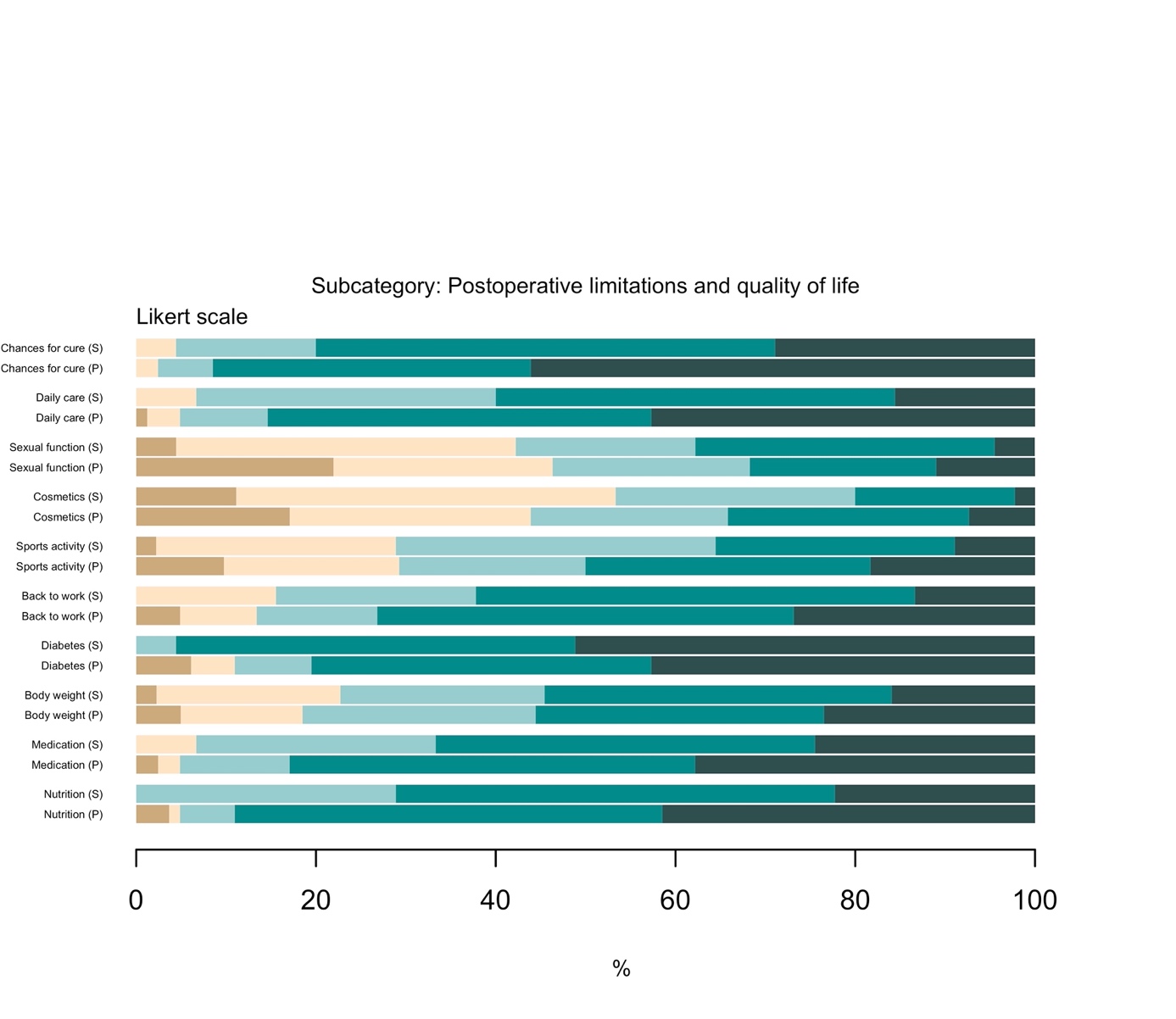


**Figure S4.** Survey results of single items compared between patients pre- and postoperatively

(A) Subcategory: Indication, timing and alternatives. *Abbreviations*: 1, preoperative survey results of patients; 2, postoperative survey results of patients; Indication, Timing, Alternatives, Prehab, Companion; refer to items nr. 1-5, Tab. S2.


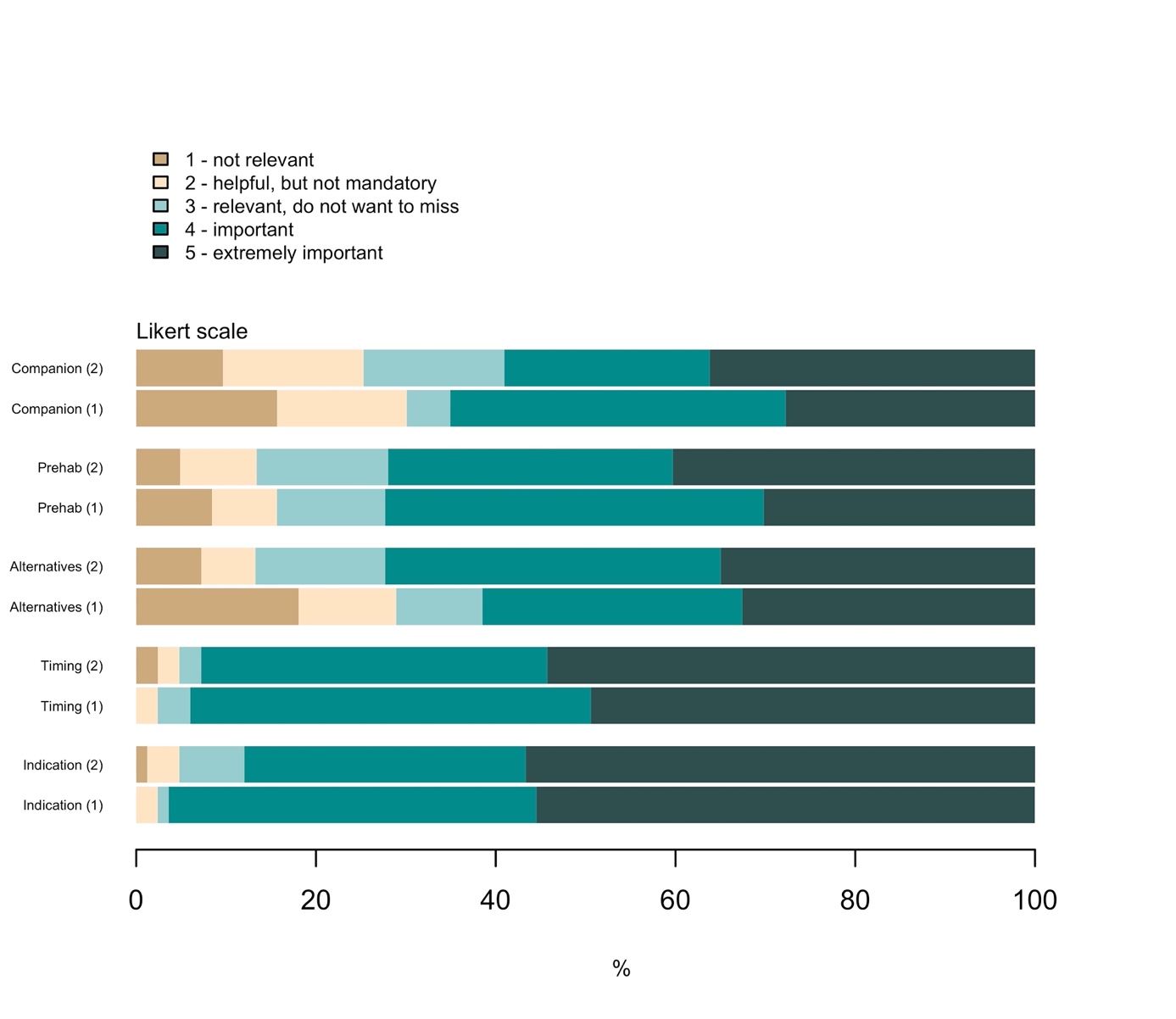


(B) Subcategory: Description of operation technique.

*Abbreviations*: *Abbreviations*: 1, preoperative survey results of patients; 2, postoperative survey results of patients; Anatomy, Drawing, Video, Approach, Preserving, Incision, Duration, Transfusion, Anaesthesia, Hospital stay, ICU stay, Nutrition, Mobilization, Ward design; refer to items nr. 6-19, Tab. S2.


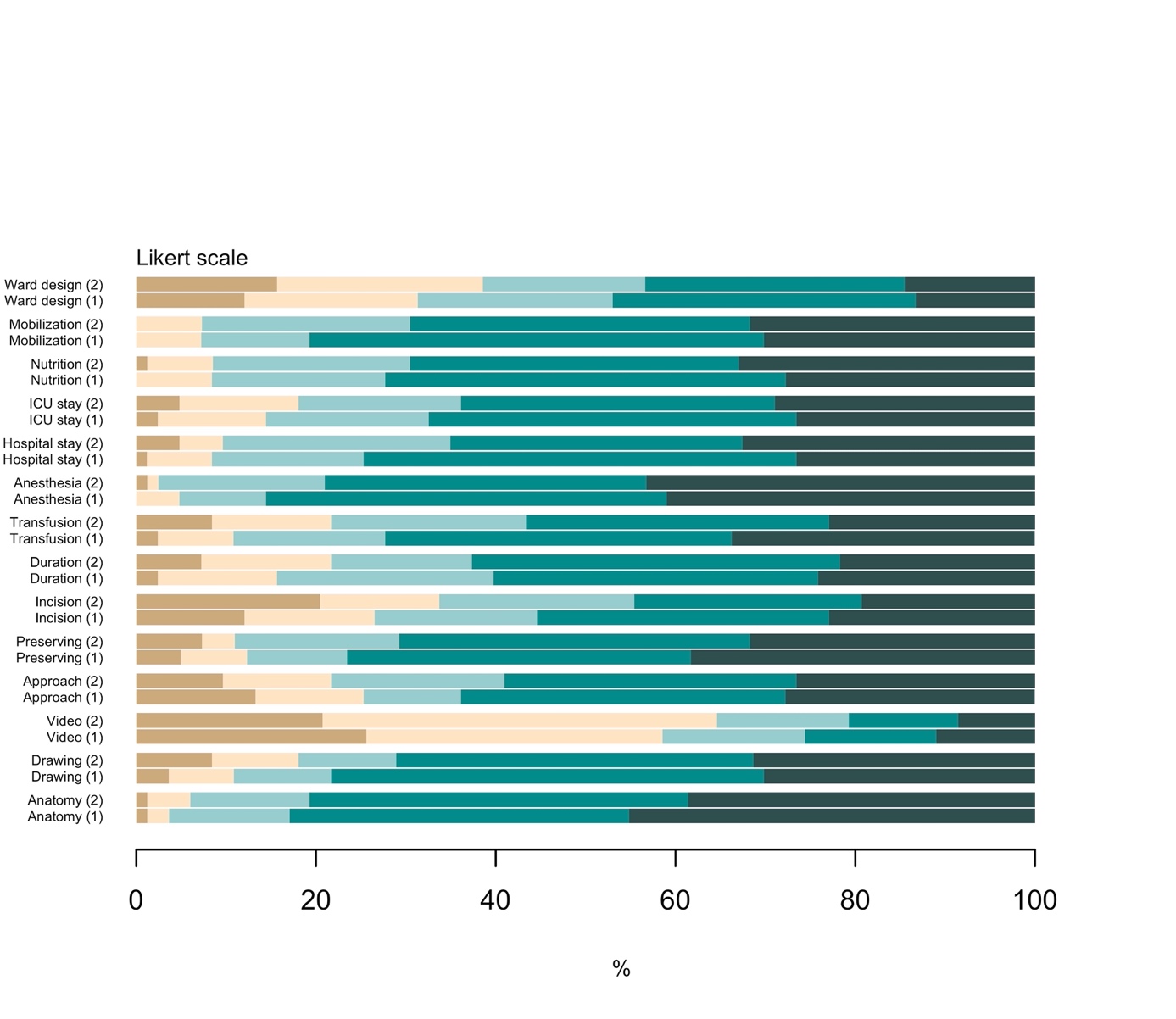


(C) Subcategory: Morbidity and Mortality.

*Abbreviations*: *Abbreviations*: 1, preoperative survey results of patients; 2, postoperative survey results of patients; Complications, Re-OP, Intervention, Infection, DVT/PE, NG tube, Pain, Intraop. problem, Stoma, MRGN/MRSA, Covid-19, Mortality; refer to items nr. 20-31, Tab. S2.


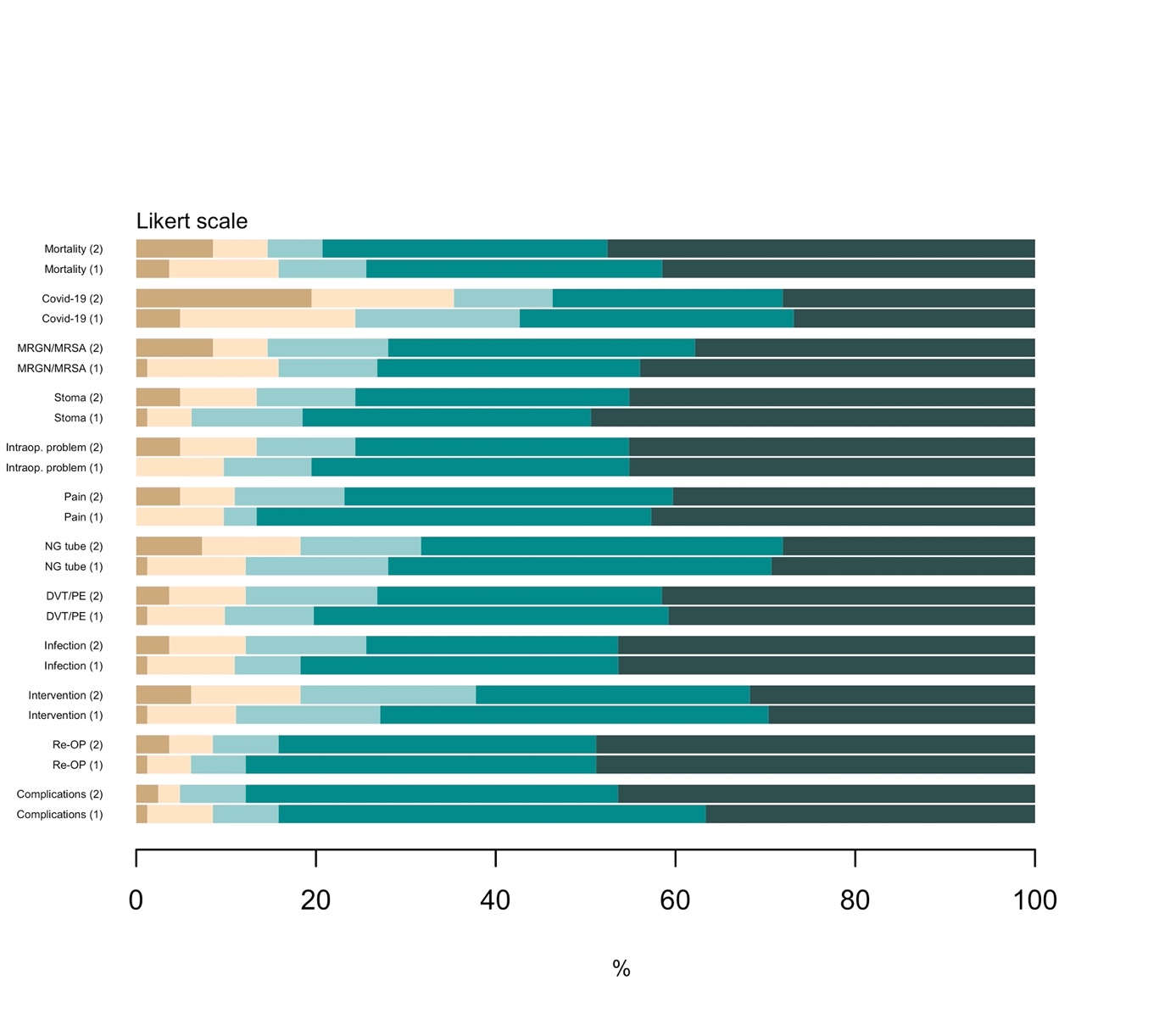


(D) Subcategory: Centre quality, volume and expertise.

*Abbreviations*: *Abbreviations*: 1, preoperative survey results of patients; 2, postoperative survey results of patients; Centre expertise, Hospital volume, Surgeon volume, Certification, Senior surgeon, 2^nd^ opinion, Equipment; refer to items nr. 32-38, Tab. S2


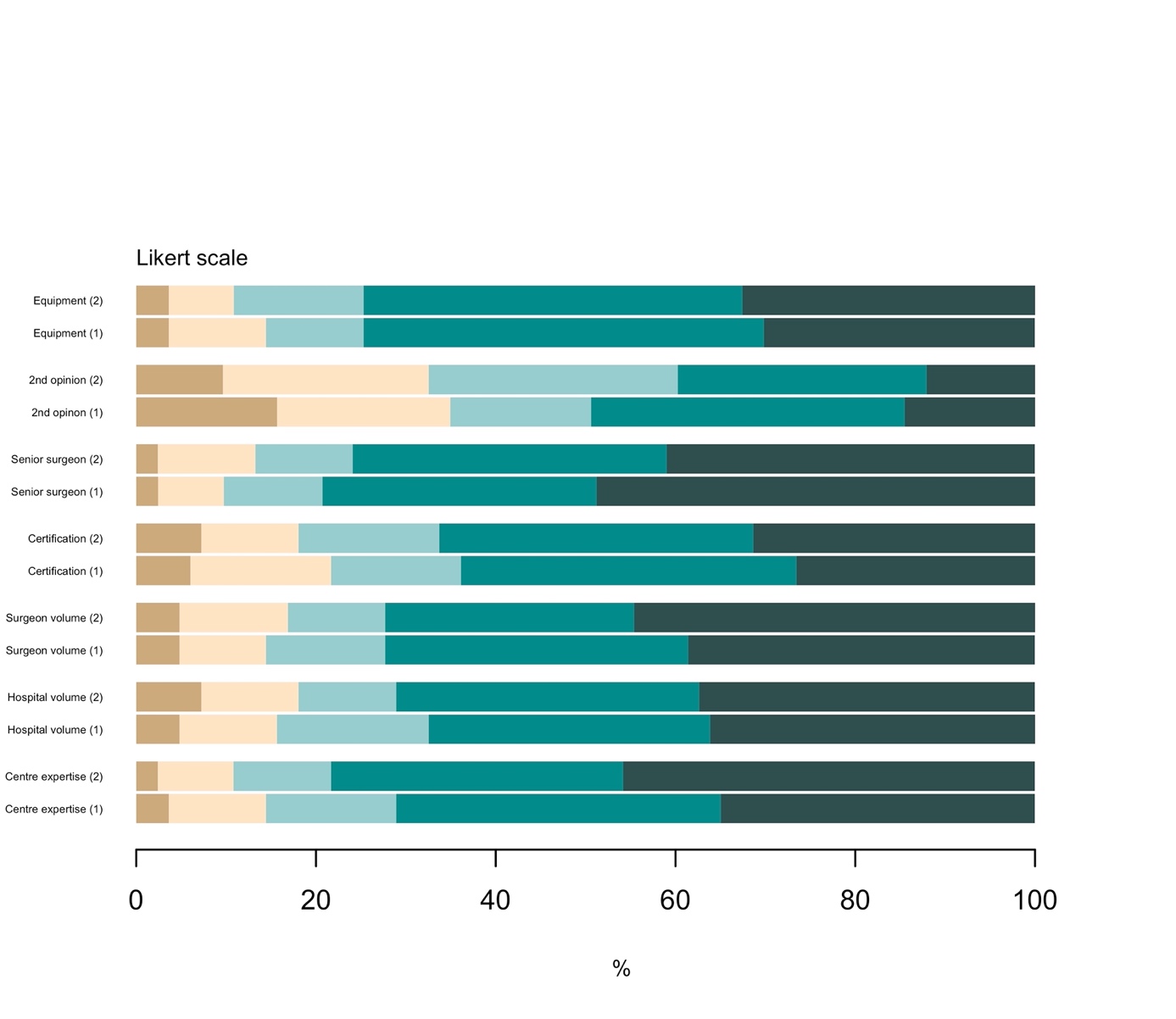


(E) Subcategory: Adjuvant treatment and rehabilitation.

*Abbreviations*: *Abbreviations*: 1, preoperative survey results of patients; 2, postoperative survey results of patients; Possible chemo, Chemo effects, Chemo duration, Radiation, Rehabilitation; refer to items nr. 39-43, Tab. S2


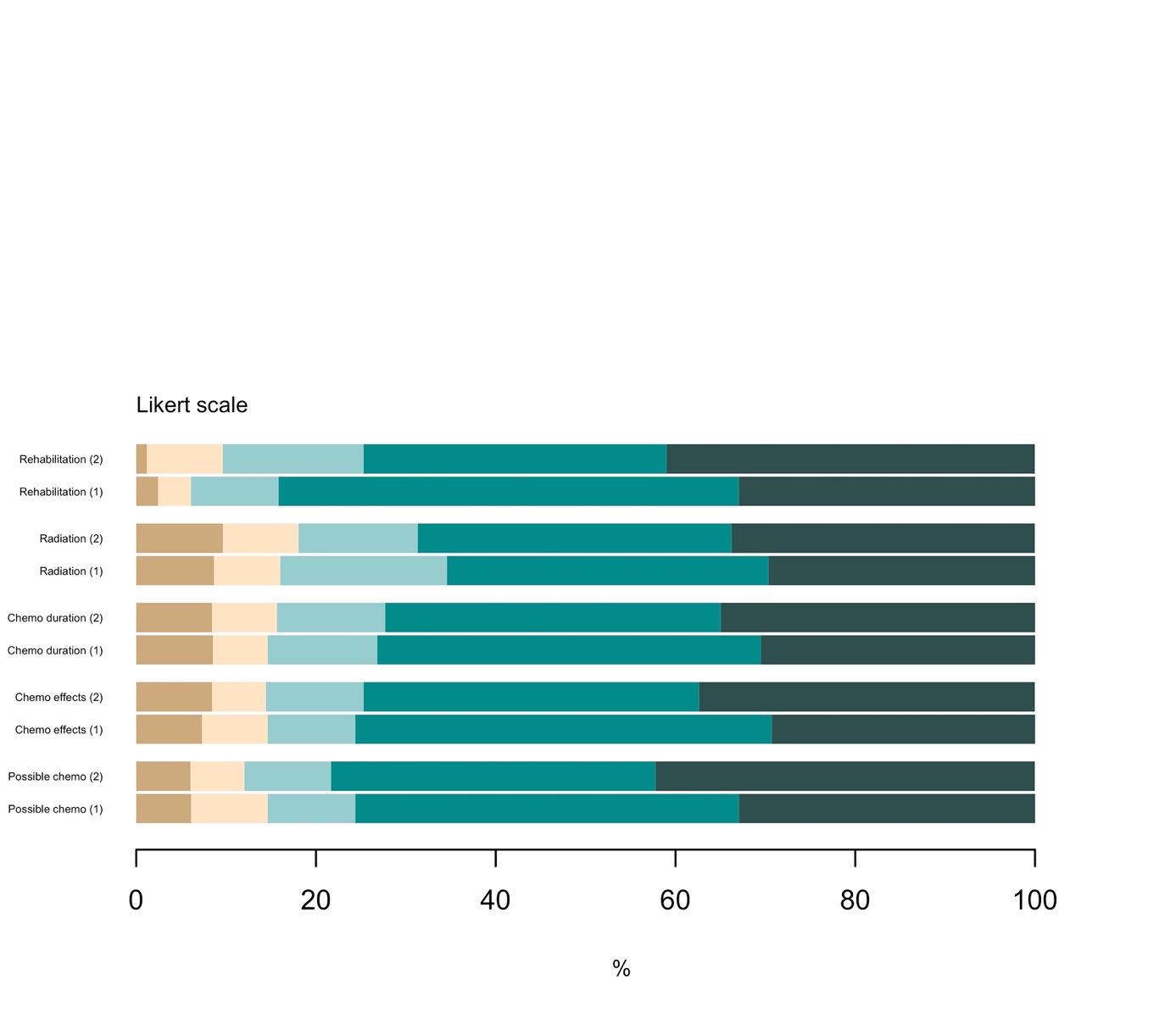


(F) Subcategory: Postoperative limitations and quality of life.

*Abbreviations*: *Abbreviations*: 1, preoperative survey results of patients; 2, postoperative survey results of patients; Nutrition, Medication, Body weight, Diabetes, Back to work, Sports activity, Cosmetics; Sexual function; Daily care, Chances for cure; refer to items nr. 44-53, Tab. S2


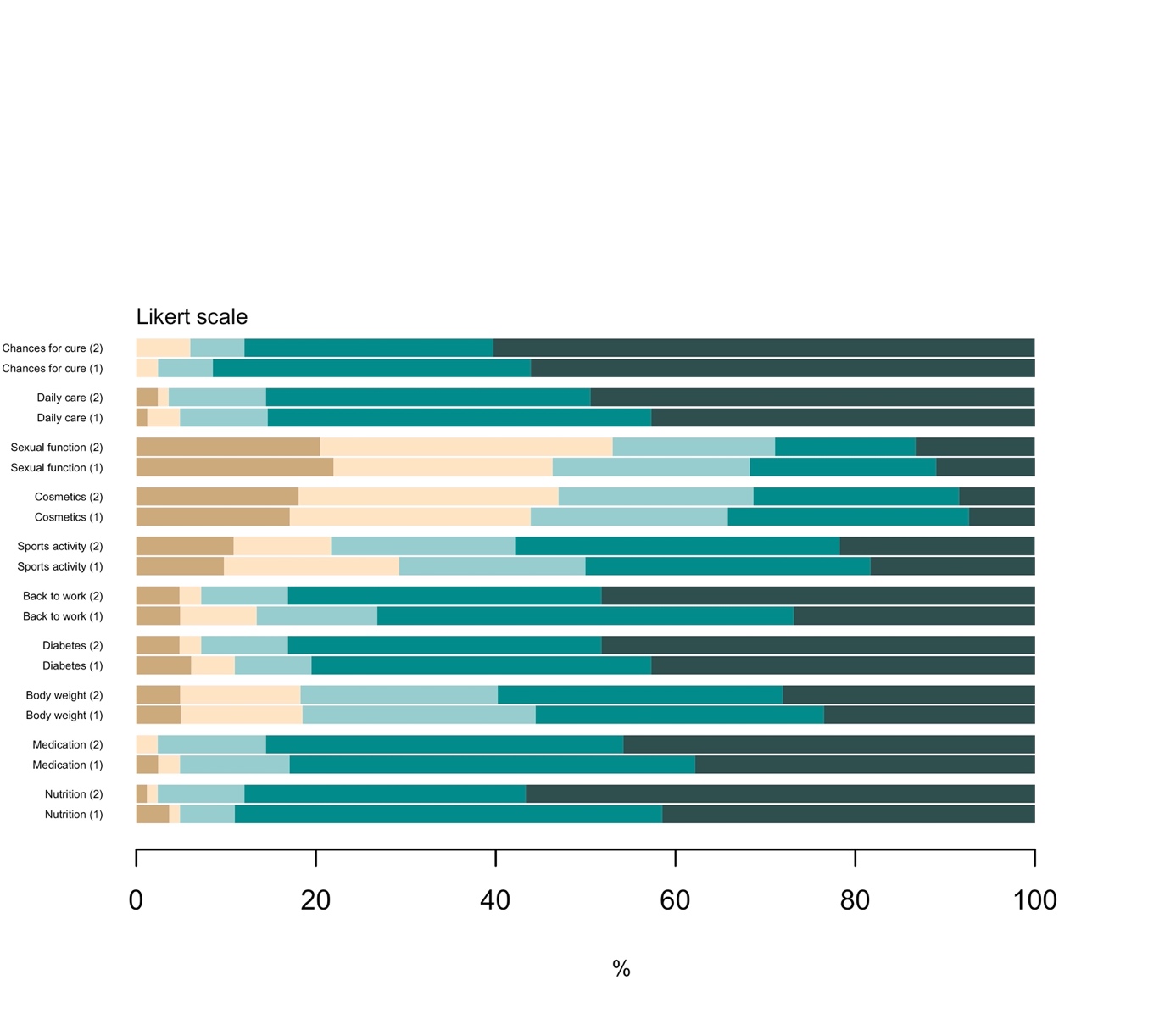


**Supplementary Tables**

**Table S1**. Content of the study survey (patient version)

| **I. Subcategory: Indication, timing and alternatives** | |
| --- | --- |
| *.* | *How important it is for you that the physician explains the following items in detail?* |
| 1. | Reasons why the operation needs to be performed (indication). |
| 2. | Urgency and timing of surgery. When should the operation be performed and why. |
| 3. | Alternatives to the operation (e.g. no treatment, medical/non-operative therapy, tissue biopsy, stent placement). |
| 4. | Information on what you can do before the operation to enhance the recovery process (e.g. fitness, nutrition, specific training program, smoking cessation). |
| 5. | Informed consent/ counselling in the presence of family members or other companions. |
| **II. Subcategory: Surgical intervention and procedure/ technique** | |
|  | *How important it is for you that the physician explains the following items in detail?* |
| 6. | Description of the anatomy before and after surgery (i.e. detailed explanation of the different steps of the operation). |
| 7. | The description is supported by a drawing or images. |
| 8. | Explanation of the operation using a video (animation). |
| 9. | Different surgical techniques (open surgery, laparoscopic / “key-hole” surgery = minimally invasive, or robot-assisted)? |
| 10. | Different variants of the operation: preservation of the stomach or partial removal of the stomach, removal or preservation of the spleen. |
| 11. | Which skin incision of the abdomen will be used. |
| 12. | Duration of the operation. |
| 13. | If blood transfusions will be necessary. |
| 14. | Pain management. |
| 15. | Length of your hospital stay. |
| 16. | Possible intensive care unit stay and for how long. |
| 17. | When will you be able to eat and drink again. |
| 18. | When will you be able to get up from bed (ambulate) again. |
| 19. | How does the ward look like, where you will stay after the operation. |
| **III. Subcategory: Morbidity and mortality** | |
|  | *How important it is for you that the physician explains the following items in detail?* |
| 20. | Explanation of the main general complications (such as bleeding, wound healing disorders, or leakage of digestive fluids into the abdominal cavity). |
| 21. | Necessity of a re-operation due to severe complications, possibly including the resection of the entire pancreas. |
| 22. | Potential computed tomography (CT) scans/examinations in the days following surgery, possibly with CT-navigated fluid aspiration in the abdominal cavity and/or transcutaneous placement of a drain. |
| 23. | Infection with bacteria and development of a blood infection (sepsis). |
| 24. | Development of thrombosis or pulmonary embolism. |
| 25. | Vomiting, potentially followed by a nasogastric tube placement. |
| 26. | How pain will be managed after the operation |
| 27. | Injury of blood vessels and other organs during the operation, which may necessitate expanding the extent of the originally planned resection. |
| 28. | Possible expansion of the operation, which may require the creation of a stoma. |
| 29. | Infection with multidrug-resistant bacteria (hospital-acquired infections) or pathogens leading to diarrhea. |
| 30. | Infection with COVID-19 during the hospital stay. |
| 31. | The fact that you may die during or after the operation. |
| **IV. Subcategory:** **Centre quality, volume and expertise** | |
|  | *How important it is for you that the physician explains the following items in detail?* |
| 32. | Description of the experience of the centre. |
| 33. | How often the procedure is performed at the centre (number of comparable operations). |
| 34. | How often the operation is performed by the responsible surgeon (number of comparable operations) |
| 35. | If there is an official certification for the operation and centre. |
| 36. | If the programme director or the most experienced senior surgeon will perform the surgery by himself. |
| 37. | You will be informed about the possibility of seeking a second opinion at another centre and possibly be provided with a respective contact information. |
| 38. | Modern equipment/ facilities of the clinic/ hospital  . |
| **V. Subcategory:** **Adjuvant treatment and rehabilitation** | |
|  | *How important it is for you that the physician explains the following items in detail?* |
| 39. | Possible chemotherapy after the operation. |
| 40. | How chemotherapy might affect my (quality of) life. |
| 41. | How long the chemotherapy might last. |
| 42. | Potential radiation therapy after the operation. |
| 43. | Possible rehabilitation programmes after the operation. |
| **VI. Subcategory: Postoperative limitations and quality of life** | |
|  | *How do you rate the importance of postoperative changes with regard to…* |
| 44.  45. | nutrition (what food can you tolerate, what food should you avoid)?  possible requirement of drugs to improve digestion after the operation? |
| 46. | weight changes after the operation (e.g. weight loss)? |
| 47. | development of diabetes mellitus? |
| 48. | ability to work or to manage daily activities. |
| 49. | sports activities? |
| 50. | appearance/ cosmetics? |
| 51. | sexual function? |
| 52. | if you may care for yourself on your own after the operation or if you will need help from others? |
| 53. | your life expectancy and/or your chances for cure? |

**Table S2.** Patient characteristics and operative and outcome results

| **Variable** | **n (%)/median (IQR)** |
| --- | --- |
| Number of patients included (n) | 83 (100%) |
|  |  |
| Age at surgery (years) | 66.0 (59.0–74.0) |
| Sex |  |
| Male | 52 (62.7%) |
| Female | 31 (37.3%) |
| Preoperative BMI (kg/m^2^) | 26.0 (62.3–28.7) |
|  |  |
| Diagnosis |  |
| Adenocarcinoma | 60 (72.3%) |
| IPMN | 5 (6.1%) |
| Cystic lesions | 2 (2.4%) |
| Chronic pancreatitis | 7 (8.4%) |
| Neuroendocrine tumour | 7 (8.4%) |
| other | 2 (2.4%) |
|  |  |
| Previous pancreatic surgery |  |
| no | 78 (94.0%) |
| yes | 5 (6.0%) |
|  |  |
| Medical background/expertise^*^ |  |
| no | 75 (90.4%) |
| yes | 8 (9.6%) |
|  |  |
| Type of surgical intervention |  |
| Pancreatoduodenectomy | 45 (54.2%) |
| Distal pancreatectomy | 18 (21.7%) |
| Total pancreatectomy | 14 (16.9%) |
| Enucleation | 2 (2.4%) |
| Exploration | 4 (4.8%) |
|  |  |
| Type of surgical technique |  |
| open | 68 (81.9%) |
| robot assisted | 10 (12.1%) |
| laparoscopic | 1 (1.2%) |
| hybrid | 4 (4.8%) |
|  |  |
| Morbidity (CDC) |  |
| 0 | 18 (21.7%) |
| 1/2/3a | 46 (55.4%) |
| 3b/4 | 18 (21.7%) |
| 5 | 1 (1.2%) |
|  |  |
| Length of hospital stay (days) | 16.0 (10.0–23.0) |
| BMI, body-mass-index; IQR = interquartile range; IPMN, intraductal papillary mucinous neoplasm; CDC, Clavien‒Dindo classification. All complications experienced during the hospital stay after index-surgery were included.  ^*^The patients were asked if they had a medical training or degree (e.g. nurse, physician assistant or physician) | |
|  | |

**Table S3.** Characteristics of the included surgeons

|  |  |
| --- | --- |
| **Surgeon characteristics** | **n (%)/median (IQR)** |
|  |  |
| Number of included subjects | 45 (100) |
|  |  |
| Age |  |
| < 35 years | 12 (26.7%) |
| 35–50 years | 26/57.8%) |
| > 50 years | 6 (13.3%) |
| NA | 1 (2.2%) |
|  |  |
| Sex |  |
| Male | 27 (60.0%) |
| Female | 17 (37.8%) |
| Diverse | 1 (2.2%) |
|  |  |
| Position/expertise |  |
| Surgical resident | 14 (31.1%) |
| Senior surgeon | 21 (46.7%) |
| Programme director or chief position at the department | 10 (22.2%) |
|  |  |
| Performed > 20 pancreatic resections as a senior surgeon |  |
| No | 30 (66.7%) |
| Yes | 15 (33.3%) |
| NA, not available |  |

**References**

1. Dindo D, Demartines N, Clavien PA. Classification of surgical complications: a new proposal with evaluation in a cohort of 6336 patients and results of a survey. Ann Surg. 2004 Aug;240(2):205-13. doi: 10.1097/01.sla.0000133083.54934.ae. PMID: 15273542; PMCID: PMC1360123.
